# Oleogels Based on a Vegetable Oil Blend Structured by a Binary Beeswax–Monoglyceride System

**DOI:** 10.3390/foods15162820

**Published:** 2026-08-13

**Authors:** Indira Temirova, Vural Gökmen, Svetlana Kamanova, Aidyn Igenbayev, Daulet Aitmukhambetov, Sayagul Tazhina, Alexsandr Borovskiy, Dina Khamitova, Kadyrzhan Makangali, Gulnazym Ospankulova

**Affiliations:** 1 Department of Food Technology and Processing Products, Technical Faculty, Saken Seifullin Kazakh Agrotechnical University, Zhenis Avenue, 62, Astana 010000, Kazakhstan; indira_t85@mail.ru (I.T.); kamanovasveta@mail.ru (S.K.); aidyn_mamyt@mail.ru (A.I.); daulet-kerei@mail.ru (D.A.); sayagultazhina@gmail.com (S.T.); a.yu.borovskiy@gmail.com (A.B.); dina.khamitova@nu.edu.kz (D.K.); k.makangali@kazatu.edu.kz (K.M.); 2 Regional Technological Park QazAgroOrganic LLP, Zhenis 62b, Astana Z11F9K5, Kazakhstan; 3 Food Quality and Safety (FoQuS) Research Group, Department of Food Engineering, Hacettepe University, Ankara 06800, Turkey; vgokmen@hacettepe.edu.tr; 4 Aron R&D Co. LLP, Akmeshit 5, Astana 010000, Kazakhstan

**Keywords:** oleogels, blended oil, beeswax, monoglyceride, oleogelators

## Abstract

The aim of this study was to produce oleogels based on a vegetable oil blend (high-oleic sunflower, safflower, and linseed oils at a ratio of 60:30:10, respectively) structured with a binary beeswax–monoglyceride system (1:1), as well as to examine the effect of the total structuring agent concentration (3, 6, 9 and 12% (*w*/*w*)) on the textural, oil-binding, microstructural, thermal, and rheological characteristics of the resulting oleogels, in order to determine the rational total concentration of the binary structuring agent system. The structuring capacity of the binary system was concentration-dependent: at 6 and 9% (*w*/*w*), the hardness of the oleogels exceeded the values of the corresponding single-component systems, whereas at 12% (*w*/*w*), no further increase in hardness was observed compared with the monoglyceride oleogel. Increasing the concentration altered crystal morphology: at 6 and 9% (*w*/*w*) a more uniform, finely dispersed structure was formed, whereas at 12% (*w*/*w*), crystal coarsening and elongation were noted. Overall, a total concentration of 6% (*w*/*w*) was considered rational, providing the required balance of characteristics at a relatively low structuring agent content. The resulting oleogel may be regarded as a potential fat base for further research on the development of spreads.

## 1. Introduction

Oils and fats are widely used in the food industry and are significant components of the human diet. Owing to the presence of fats, products acquire characteristic textural and flavor features, as well as functional properties such as plasticity and hardness [1]. One of the fat products consumed in the everyday diet is spreads, the manufacture of which uses solid fats that are mostly characterized by an elevated content of saturated fatty acids (SFAs) and, in certain cases, fatty acid trans-isomers [2].

A promising direction in the oil-and-fat industry is the use of structured vegetable oils (oleogels), in which liquid vegetable oil is retained within the crystalline network of a structuring agent, thereby acquiring a semi-solid consistency. Oleogels are regarded as an effective alternative to solid fats for reducing the content of SFAs and TFAs [1,3]. In addition, they offer the advantage of being able to preserve the fatty acid composition and nutritional profile of the original vegetable oils, which are rich in unsaturated fatty acids, thereby enhancing their nutraceutical value and the functional characteristics of food products [4,5].

The relevance of research into the structuring of vegetable oils is confirmed by previously published scientific works, in which oleogels based on vegetable oils and various structuring agents were used as a partial replacement for saturated vegetable oil in chocolate spreads, in the baking of confectionery and bakery products, in the production of margarine, and in many other products [4,6,7].

Despite existing scientific works obtaining oleogels based on various vegetable oils, such as soybean oil [8], rapeseed oil [9], rice bran oil [10], olive oil [11], and sunflower oil [12], there is currently insufficient research devoted to the production of oleogels based on blended vegetable oils balanced in their ω-6 to ω-3 ratio and structured with binary structuring agents.

According to studies, excessive consumption of SFAs and TFAs contributes to an increase in the level of total cholesterol and low-density lipoproteins, which increases the likelihood of disorders in the cardiovascular system and leads to disease [13,14,15].

Therefore, restrictions on the content of TFAs in food products have been introduced in many countries. The World Health Organization (WHO) recommends that their share in the daily diet should not exceed 1% of the total energy value, and that the consumption of SFAs should be less than 10%. In addition, an important role is noted for the balanced consumption of the essential fatty acids, omega-6 and omega-3, within the recommended range of 5:1 to 10:1 [16].

The results of previously conducted studies indicate that replacing saturated fats with unsaturated vegetable oils has a positive effect on health indicators and increased life expectancy [17,18,19]. However, vegetable oils obtained from a single raw material source often do not provide an optimal balance of functional and nutritional properties, which gives rise to the need for their blending, particularly in the production of oleogels [20,21,22].

The nature of the interaction between the structuring agent and the oil phase plays an important role in the formation of the oleogel structure. Since the oil phase is the basis of the oleogel, its composition and properties largely determine the features of structure formation and the characteristics of the resulting oleogels. Previously conducted studies have established that the fatty acid composition of vegetable oils, which is one of their key characteristics, is regarded as an important factor capable of influencing the structuring process and the properties of the resulting oleogels [23]. Previous studies have demonstrated that minor constituents of vegetable oils and various bioactive compounds can affect the structuring behavior of oleogel systems [24,25].

In the work of Frolova et al. [26], the authors showed that the nature of the oil phase exerts a significant influence on the characteristics of oleogels. Under identical technological conditions, different oils structured with beeswax give rise to the formation of crystals with different morphologies. In the study of Gravelle et al. [23], the authors established that oleogels structured with ethylcellulose were stronger with linseed oil than oleogels made from soybean and rapeseed oils. In the work of Ferro et al. [27], it was established that the properties of the oil phase affect the self-assembly of monoglycerides and the formation of the crystalline network of oleogels, determining their microstructure, textural characteristics and thermal behavior.

The process of structuring vegetable oils also largely depends on the composition of the triglycerides in the oil, with differences observed between systems based predominantly on long-chain and medium-chain triglycerides, which is reflected in the formation of the crystalline structure, particularly in the density of and distance between the crystalline elements [28].

Overall, these results indicate that the oil phase acts as an important component influencing the structuring process and the final properties of oleogels [29].

In addition to the composition of the oil phase, the structuring agent used has a substantial influence on the properties of oleogels. Despite the diversity of structuring agents used for the gelation of liquid vegetable oils, most studies have focused on single-component systems employing natural waxes, monoglycerides, ethylcellulose and other structuring agents [30,31,32,33], whereas binary structuring agents remain less studied, although they offer considerable potential for improving the properties of oleogels and overcoming the limitations of the individual structuring agents [34,35,36].

Among the known structuring agents, beeswax and monoglycerides are of particular interest. Their combined use is regarded as a promising approach to obtaining oleogels, since the combination of these structuring agents may promote the formation of a stronger and more stable structure than the use of each structuring agent individually [36,37].

In this regard, it is of interest to investigate the effect of the total content of the binary structuring agent system on the properties of oleogels produced from a blended oil.

Despite a considerable number of studies devoted to oleogels based on beeswax, monoglycerides and their combinations, the effect of the total concentration of the binary system at a fixed component ratio on the properties of oleogels produced from a multicomponent vegetable oil blend has been insufficiently studied. Most published works consider individual oil types, single-component structuring agents, or binary systems at a limited number of concentrations.

In view of the above, the aim of this study was to produce oleogels based on a vegetable oil blend (high-oleic sunflower, safflower and linseed oils at a ratio of 60:30:10, respectively) structured with a binary beeswax–monoglyceride system (1:1), and to examine the effect of the total structuring agent concentration (3, 6, 9 and 12% (*w*/*w*)) on the textural, oil-binding, microstructural, thermal and rheological characteristics of the resulting oleogels, in order to determine the rational total concentration of the binary structuring agent system.

## 2. Materials and Methods

### 2.1. Materials

Vegetable oils, such as refined high-oleic sunflower oil (HOSO), unrefined safflower oil, and unrefined linseed oil, were purchased at local supermarkets: High-oleic sunflower oil (ASTON, Rostov-on-Don, Russia), unrefined safflower oil (Donya, Shymkentmay JSC, Shymkent, Republic of Kazakhstan), and unrefined linseed oil (Healthy Oil Products, Zharkol007 LLP, Kostanay, Republic of Kazakhstan). Beeswax (BW) was provided by a beekeeping farm in the East Kazakhstan region, with a melting point of 62–65 °C; distilled monoglycerides (E471) of the Ekomul MG 95 HP brand (Ecolex Sdn. Bhd., Port Klang, Malaysia) with a monoglyceride content of at least 95% were used. All chemical reagents used in this study were of analytical grade and purchased from Sigma-Aldrich (Burlington, MA, USA).

### 2.2. Preparation of the Blended Oil

The composition of the blended oil was calculated using the linear programming method of Yadav et al. [38], in order to obtain the recommended range for the ratio of n-6 to n-3 polyunsaturated fatty acids of 5:1–10:1, taking into account the modern principles of rational nutrition recommended by the WHO [16]. The appropriate amounts of vegetable oils were weighed and stirred in a laboratory reactor LR 1000 basic (IKA-Werke GmbH & Co. KG, Staufen im Breisgau, Germany) at a speed of 150 rpm for 15 min (at 30 °C) to ensure uniform distribution, cooled to 25.0 ± 0.5 °C and stored at 4 °C until analysis.

### 2.3. Preparation of the Oleogel from the Oil Blend

Oleogels were prepared according to the method of Dimakopoulou-Papazoglou et al. [29], with some modifications. To obtain the oleogels, binary structuring agent systems of beeswax–monoglyceride at a fixed mass ratio of 1:1 (*w*/*w*) were used. The choice of this ratio was based on the results of previously published works, in which its effectiveness in forming a stable gel-like structure was demonstrated [39]. The blended oil was heated to 80 °C, the structuring agents were added at concentrations of 3%, 6%, 9% and 12% (*w*/*w*), and the mixture was stirred until complete dissolution to obtain a homogeneous dispersion. The mixture was then mechanically stirred for 20 min in an LR 1000 basic laboratory reactor at 150 rpm. The resulting oleogels were cooled at 25.0 ± 0.5 °C for 60 min and subsequently at 4 °C for 24 h. All measurements were performed after holding the oleogel samples at 25.0 ± 0.5 °C for 2 h.

### 2.4. Fatty Acid Composition Analysis

The fatty acid composition of the starting vegetable oils and the blended oil was determined by gas chromatography with mass spectrometric detection (GC-MS) following prior conversion to fatty acid methyl esters (FAME). Sample preparation of the starting vegetable oils and blended oil was carried out in accordance with the recommendations of ISO 12966-1:2014 and ISO 12966-2:2017 [40,41], adapted to the conditions of the analytical equipment used. Fatty acid methyl esters were prepared by BF_3_-MeOH-catalyzed transesterification based on the approach described by Araujo et al. [42], with minor modifications. A weighed portion of oil of 0.10 ± 0.01 g was placed in a hermetically sealed glass vial, followed by addition of 2.0 mL of n-hexane and 2.0 mL of BF_3_-MeOH solution. The resulting mixture was vortexed for 1 min and heated at 60 °C for 20 min. After cooling to room temperature, 1.0 mL of distilled water was added to the vial. The mixture was centrifuged at 2500 rpm for 7 min, after which the upper organic layer containing the FAME was transferred to a vial for gas chromatographic analysis. FAME analysis was performed on a GCMS-QP2020 NX gas chromatograph–mass spectrometer (Shimadzu Corporation, Kyoto, Japan) equipped with an AOC-20i Plus autosampler and a CP-Sil 2560 capillary column (100 m × 0.25 mm × 0.20 µm). Helium of 99.999% purity was used as the carrier gas. The injection volume was 1.0 µL with a split ratio of 1:40. Regarding injector parameters, the temperature was set to 250 °C, whereas interface temperature was set to 260 °C. The column oven temperature program involved an initial hold at 100 °C for 5 min, followed by ramping to 210 °C at 4 °C/min with an 8 min hold. Consequently, the temperature was raised to 240 °C at 10 °C/min and held at this temperature for 16.5 min.

FAME identification was performed based on retention times using a 37 FAME Mix standard (Supelco, Merck, Darmstadt, Germany). For additional confirmation of identification, mass spectra were matched against the NIST library. The relative content of fatty acids was calculated by the peak area normalization method for all identified peaks and expressed as a percentage of the total area of detected FAME. From the resulting profile, the total content of saturated, monounsaturated, and polyunsaturated fatty acids, as well as the ω-6/ω-3 ratio, was additionally determined. The calculated oxidizability value (COX) of the fat phase for the oils and the blended oil was calculated using Equation (1), as proposed by Fatemi & Hammond [43].

(1) $$COX=\frac{C18:1+(10.3\times C18:2)+(21.6\times C18:3)}{100}$$

### 2.5. Determination Hardness

The hardness of the oleogels was determined using a TA.XTPlusC texture analyzer (Stable Micro Systems, Godalming, UK) equipped with a 45° conical probe (P/45C; maximum diameter, 30 mm). The probe was inserted into the sample at a depth of 15 mm with a test speed of 2 mm/s. Hardness was defined as the maximum force recorded during penetration and was expressed in newtons (N).

### 2.6. Determination of Oil-Binding Capacity

The oil-binding capacity was determined by centrifugation on an OHAUS Frontier™ 5000 Series Multi Pro FC5816R refrigerated centrifuge (Nänikon, Switzerland) according to the method described in Yılmaz & Öğütcü [44], with some modifications. Before analysis, the oleogel samples were held for 24 h at room temperature to reach an equilibrium state. A sample portion of oleogel weighing 1.000 ± 0.001 g was placed in pre-weighed centrifuge tubes and reweighed. The tubes were then centrifuged at 11,000× *g* for 10 min. The tubes were turned over onto filter paper to remove the filtered-out oil. After centrifugation, the oil-binding capacity was calculated using Formulas (2) and (3).

(2) $$\%Released\;oil=\frac{Mass\;of\;expressed\;oil(g)}{Total\;mass\;of\;sample\;(g)}\times 100$$

(3) %OBC=100−%released oil

### 2.7. Inverse Vial Test on Oleogel Structure Formation

After stabilization at 4 °C for 24 h, the sample tubes were inverted by 180° and maintained in the inverted position for 30 s. Samples were considered to have successfully formed an oleogel when no flow, mass displacement, or structural collapse was observed under the influence of gravity.

### 2.8. Determination of Thermal Properties of Oleogels

The thermal properties of the oleogels were evaluated using a DSC 300 Caliris Supreme differential scanning calorimeter (NETZSCH-Gerätebau GmbH, Selb, Germany) with a connected cooling device (IntraCooler) according to the method of Okuro et al. [45]. Accordingly, the 5 mg of sample was placed to hermetically sealed disposable aluminum pan, and the empty one was used as a reference. Initially samples were held at 0 °C, with consequent heating to 100 °C and held under isothermal conditions for 5 min. Then, they were cooled to 0 °C (cooling stage), held under isothermal conditions for 30 min, and finally heated again to 100 °C. Heating and cooling rate parameters were carried out at a constant rate of 5 °C·min^−1^. The data were processed using Proteus software, version 9.0.4.

### 2.9. Determination of Microstructural Properties of Oleogels

The microstructure of the oleogels was investigated by light microscopy using an Axioscope 5 upright light microscope (Carl Zeiss Microscopy GmbH, Jena, Germany) according to Jeong & Oh [36], with some modifications. Micrographs were obtained in EPI DF mode, corresponding to dark-field observation under reflected light. Before analysis, a small amount of the sample was applied as a thin layer onto a glass slide and distributed evenly over the surface. Finally, the micrographs were analyzed using Zen 3.0 software (blue edition), Carl Zeiss GmbH, with a camera (Axiocam 305 color).

### 2.10. Fourier-Transform Infrared Spectroscopy (FTIR)

Infrared spectroscopic analysis of the oleogels was carried out on a Shimadzu IR Spirit-X FTIR spectrometer (Shimadzu Corporation, Kyoto, Japan) equipped with a diamond Quest ATR attachment (Specac Ltd., Orpington, UK), according to Dimakopoulou-Papazoglou et al. [46]. Before measurement, the samples were held at 25 °C for 3 h, after which a small amount of oleogel was applied to the ATR plate. The spectra were recorded at room temperature in the range of 4000–500 cm^−1^ with a resolution of 4 cm^−1^; 32 scans were performed for each spectrum. Before the analysis of each sample, a background spectrum of air was recorded, which was then automatically subtracted from the sample spectrum. Five spectra were obtained for each experimental variant.

### 2.11. Determination of Apparent Viscosity

The apparent viscosity of the oleogels was determined using a Brookfield rotational viscometer (model DVNext, AMETEK Brookfield, Middleboro, MA, USA) equipped with a Helipath stand (HPT00) and a T-D T-bar spindle. Measurements were carried out at 28.0 ± 0.5 °C over a rotational speed range of 2 to 12 rpm in 2 rpm increments (sequence: 2, 4, 6, 8, 10 and 12 rpm). At each speed, the spindle was rotated for 30 s, and viscosity values were recorded every 10 s. Only results obtained at a torque of 10–100% of the instrument full scale were taken into account. Measurements were performed in triplicate and the results were expressed in Pa·s.

### 2.12. Statistical Analysis

All measurements were carried out in at least triplicate. The results were presented as the mean value with an indication of the standard error of the mean. Statistical data processing was performed using Minitab 22 software. One-way analysis of variance (ANOVA) was used to compare mean values, and the subsequent assessment of differences between variants was carried out using Tukey’s test. Differences were considered significant at a significance level of *p* < 0.05.

## 3. Results and Discussion

### 3.1. Free Fatty Acid Profiles of Vegetable Oils and Liquid Quality Indicators

The results of the investigation of the fatty acid composition of the original vegetable oils (high-oleic sunflower, unrefined safflower and unrefined linseed) and of the blended oil obtained by the linear programming method in a ratio of 60% high-oleic sunflower oil, 30% unrefined safflower oil and 10% unrefined linseed oil are presented in Table 1.

The choice of vegetable oil was driven by the need to form oleogels with a more favorable ratio of fatty acid composition, especially an improved ratio of omega-6 to omega-3 polyunsaturated fatty acids.

Thus, high-oleic sunflower oil is characterized by a high content of oleic acid (ω-9), which provides oxidative stability and stability of the fat system [47]. Safflower oil is a rich source of linoleic acid (ω-6), an essential polyunsaturated fatty acid, which determines its value as a dietary source of ω-6 fatty acids [48]. Linseed oil is characterized by a high content of α-linolenic acid (18:3, ω-3), an essential polyunsaturated fatty acid, and is a valuable source of ω-3 fatty acids, which are of major importance in human nutrition [49]. The results of the major free fatty acids of high-oleic sunflower oil, unrefined safflower oil and unrefined linseed oil corresponded to the ranges provided for in CODEX STAN 210 for their respective vegetable [50].

Analysis of the data presented in Table 1 revealed that, in HOSO, the dominant fatty acid was oleic acid (80.29%), and in safflower oil, it was linoleic acid (77.42%). The HOSO and unrefined safflower oil contained very little linolenic acid, at 0.18% and 0.09%, respectively, whereas linseed oil was a rich source of linolenic acid (49.92%). According to Table 1, the ω-6:ω-3 ratio in high-oleic sunflower, unrefined safflower and unrefined linseed oils was 45:1, 860:1 and 0.32:1, respectively. The resulting blend of vegetable oils was characterized by an improved ratio of ω-6 to ω-3 polyunsaturated fatty acids at 6:1, which is within the range of 5:1–10:1 recommended by the World Health Organization (WHO) [16].

Analysis of the fatty acid group composition revealed substantial differences between the individual oils. High-oleic sunflower oil was characterized by a predominance of monounsaturated fatty acids (MUFAs), the content of which was 80.72%, with a relatively low proportion of polyunsaturated fatty acids (PUFAs) at 8.30%. In safflower oil, by contrast, PUFAs predominated (77.51%), whereas the MUFA content was 12.00%. Linseed oil was likewise characterized by a high PUFA content (65.76%), being a valuable source of ω-3 fatty acids. Blending yielded an oil composition with a more balanced fatty acid profile, containing 53.63% MUFAs, 34.59% PUFAs and 10.97% saturated fatty acids (SFAs). The substantial content of essential PUFAs accounts for the nutritional value of the resulting oil composition, whereas the predominance of MUFA may contribute to its improved oxidative stability.

The MUFA/SFA ratio is among the significant lipid quality indicators and is used to assess the nutritional value of oils, since it reflects the balance between monounsaturated and saturated fatty acids [51,52].

The MUFA/SFA ratio revealed significant differences between the oils studied. The highest value of the indicator was established for HOSO (7.72), which indicates a predominance of monounsaturated fatty acids and potentially higher oxidative stability of the lipid system. Safflower and linseed oils were characterized by lower MUFA/SFA values (1.21 and 1.15, respectively), which is due to their high content of polyunsaturated fatty acids and may indicate a greater tendency towards oxidative changes. For the blended oil, the value of the indicator was 4.88, reflecting a more balanced ratio of monounsaturated to saturated fatty acids. Such a ratio is favorable from the point of view of nutritional value and may be regarded as a factor potentially increasing the resistance of the oil to oxidation.

A similar blending effect has been reported in other studies. Thus, in one study [53], the blending of safflower and linseed oils increased the proportion of ω-3 fatty acids and adjusted the ω-6/ω-3 ratio. In the work of Romanić et al. [54], the addition of linseed oil to sunflower oil altered the contents of linoleic and α-linolenic acids. The results of these studies show that the selection of oils and their ratios makes it possible to purposefully regulate the fatty acid profile of the resulting compositions.

The calculated oxidizability (COX) value, used to assess the potential tendency of an oil towards autoxidation, depends substantially on the fatty acid profile of the oils studied. A decrease in the COX index indicates an increase in the oxidative stability of the oil [55]. Based on the calculated COX values (Table 1), it can be concluded that the oils had different values of this indicator. The lowest COX value was obtained for HOSO (1.68). This is explained by the HOSO and the comparatively small proportion of polyunsaturated fatty acids, so such an oil can be considered more resistant to oxidative changes. For safflower oil, the COX value was noticeably higher and amounted to 8.11, which is associated with the predominance of linoleic acid. The highest calculated oxidizability was characteristic of linseed oil (12.59). Such a result is to be expected, since linseed oil contains a significant amount of α-linolenic acid, which is more readily involved in oxidation processes than oleic and linoleic acids. The data obtained are consistent with previously conducted studies, in which the calculated COX value increased as the proportion of polyunsaturated fatty acids increased [56].

For the blended oil, the COX value was 4.64. It was higher than that of HOSO but significantly lower than that of safflower and linseed oils. Thus, the blending of oils made it possible to partially reduce the potential oxidizability of the fat phase through the inclusion of high-oleic sunflower oil. At the same time, the presence of safflower and linseed oils ensures the supply of essential polyunsaturated fatty acids. Therefore, the resulting blend can be regarded as a more balanced oil base in which an improved fatty acid composition is combined with a moderate calculated tendency towards oxidation.

### 3.2. Hardness and Oil-Binding Capacity of Oleogels

To determine the rational concentration of binary structuring agents for the formation of oleogels, which may serve as potential fat systems for subsequent use in spread formulations, the prepared samples were analyzed in terms of their hardness and oil-binding capacity.

Initially, single-component oleogels based on beeswax and monoglyceride at concentrations of 3%, 6%, 9%, and 12% (*w*/*w*) were investigated, which allowed the effect of the structuring agents on hardness and oil-binding capacity to be evaluated and the results obtained to be subsequently compared with the characteristics of the binary BW + MG system. The results of the study of single-component oleogels are presented in Table 2.

The obtained data show that an increase in the concentration of structuring agents led to an increase in the hardness of the single-component oleogels. For beeswax-based samples, hardness increased from 0.08 to 4.75 N, whereas for monoglyceride-based oleogels, it increased from 1.02 to 6.28 N. Across the entire concentration range, monoglyceride-based oleogels were harder than the beeswax-based samples. This difference is likely attributable to the ability of monoglycerides to form a denser lamellar structure within the oil phase, which enhances the resistance of the gel matrix to mechanical deformation. The obtained results are consistent with the findings of Malvano et al. [57], who, when comparing food-grade oleogels based on beeswax and monoglyceride, also reported more pronounced hardness in monoglyceride-based oleogels. However, hardness values depend not only on the type and concentration of the structuring agent, but also on the conditions of oleogel preparation and on the composition of the oil phase, including the ratio of saturated, mono- and polyunsaturated fatty acids, the triacylglycerol distribution and the content of minor components capable of affecting the solubility of the structuring agent and intermolecular interactions [28].

**Figure 1:**
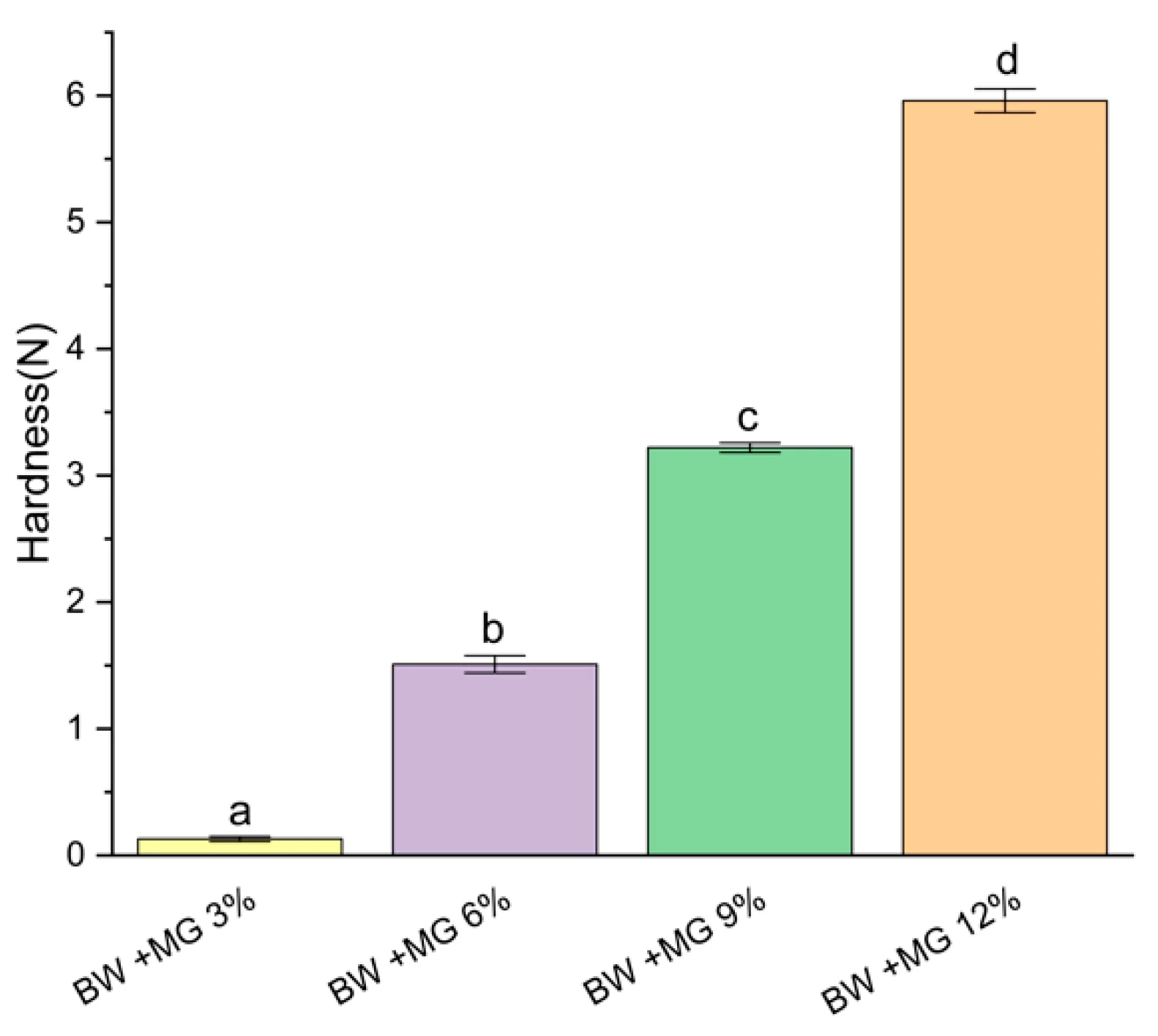
Hardness values of the oleogels. Values are presented as mean ± standard deviation (*n* = 3); different letters within the same structuring agent system indicate significant differences between concentrations (*p* < 0.05).

Figure 1 presents the hardness values of oleogels based on the binary BW + MG (1:1) system at total structuring agent concentrations of 3, 6, 9, and 12% (*w*/*w*).

According to the data given in Figure 1, a substantial increase in the hardness of the samples was observed with increasing concentration of structuring agents. The maximum hardness was noted at a concentration of 12%—5.96 N—whereas the minimum values were noted for the sample with a 3% structuring agent content—0.13 N. At the same time, the most acceptable hardness values were established for oleogels with structuring agent concentrations of 6 and 9% (*w*/*w*), amounting to 1.51 and 3.22 N, respectively. These values fall within the hardness range that provides a spreadable consistency in spreads. According to the literature, the optimal hardness for butter substitutes ranges from 1.0 to 3.3 [58], whereas lower values may result in an excessively soft structure and higher values contribute to an overly firm texture that impedes distribution of the product.

The results obtained are consistent with previously conducted studies, which state that an increase in the concentration of structuring agents promotes the strengthening of intermolecular interactions, including the formation of hydrogen bonds, which leads to the formation of a stronger and more stable crystalline network, thereby reinforcing the oleogel matrix [59,60,61].

**Figure 2:**
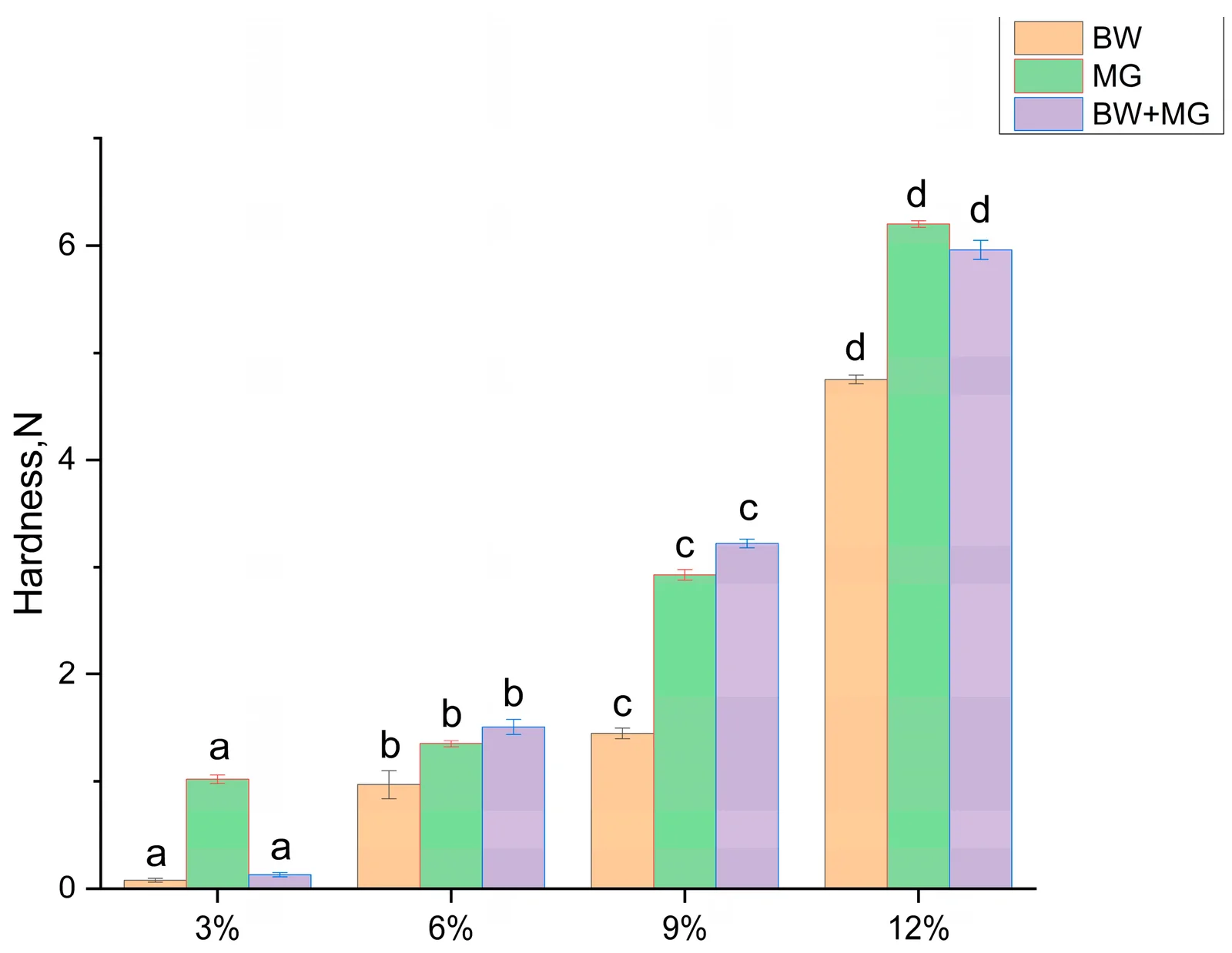
Hardness values of single-component oleogels structured with beeswax (BW) or monoglycerides (MGs) and of binary oleogels (BW + MG, 1:1) at total structuring agent concentrations of 3, 6, 9 and 12% (*w*/*w*). Values are presented as mean ± standard deviation (*n* = 3); different letters within the same structuring agent system indicate significant differences between concentrations (*p* < 0.05).

To evaluate the effectiveness of the binary system, the results obtained were compared with the hardness of single-component oleogels. At 3%, the hardness of the mixed BW + MG oleogel was 0.13 N. This value was close to that of the beeswax-based sample (0.08 N) but noticeably lower than the hardness of the monoglyceride-based oleogel (1.02 N). This is likely because the low total concentration was insufficient to form a robust structure within the oil phase. Comparative hardness values of the single-component and binary oleogels are presented in Figure 2.

As the concentration increased to 6 and 9%, the binary oleogels exhibited higher hardness compared to the single-component samples. At 6%, for instance, the hardness of the binary oleogel reached 1.51 N, whereas that of the beeswax- and monoglyceride-based oleogels was 0.97 N and 1.35 N, respectively. The same trend was observed at 9%: the hardness of the mixed oleogel was 3.22 N, exceeding the values obtained for the samples structured with beeswax alone (1.45 N) or monoglyceride alone (2.93 N).

It may be assumed that at these concentrations, the interaction between the two structuring agents contributed to the formation of a more cohesive gel network and to an increase in its resistance to mechanical deformation.

At a total structuring agent concentration of 12%, the hardness of the binary oleogel (BW + MG, 1:1) reached 5.96 N. This value exceeded that of the beeswax-only sample (4.75 N) but was somewhat lower than that of the oleogel structured solely with monoglyceride (6.28 N). Thus, the effect of the combined use of beeswax and monoglycerides on the mechanical properties of the oleogels was concentration-dependent. At a total concentration of 3% (*w*/*w*), the binary system exceeded in hardness only the beeswax-based sample. At concentrations of 6 and 9% (*w*/*w*), it provided the highest hardness values among the systems studied, whereas at 12% (*w*/*w*), the maximum hardness was established for the single-component monoglyceride oleogel.

A positive effect of the combined use of beeswax and monoglycerides has also been reported by Jeong and Oh, who established that the incorporation of glycerol monostearate into a beeswax-based system increased the hardness and oil-binding capacity of the oleogels [36]. Taken together, the present and literature data show that the structural and mechanical properties of mixed oleogels are determined not only by the total concentration of the structuring agents, but also by their ratio, their compatibility within the oil phase and their mutual influence on the formation of the crystal network.

It was established that, compared with the single-component oleogels, the binary system was most effective at concentrations of 6 and 9% (*w*/*w*). These samples possessed sufficient hardness without the formation of an excessively firm texture. Thus, the 6% oleogel sample is suitable for obtaining a soft spread, while the 9% sample is suitable for a spread of medium hardness.

Oil-binding capacity is an indicator that assesses the ability of oleogels to bind oil. This is an important indicator for assessing the quality of oleogels [62].

Oil-binding capacity of the single-component oleogels varied depending on the type of structuring agent and its concentration. In the beeswax-based samples, an increase in concentration from 3 to 12% was accompanied by an increase in oil-binding capacity from 61.20 to 99.55%. The most notable increase was already observed at 6%, when the value reached 98.10%. A further increase in concentration to 9 and 12% resulted in only a marginal change in oil-binding capacity.

In the monoglyceride-structured oleogels, oil-binding capacity likewise increased with increasing concentration, from 58.53 to 95.52%; but across the entire concentration range studied, the oil-binding capacity values remained lower than those of the beeswax-based samples.

The results of the oil-binding capacity of single-component oleogels structured separately with beeswax and monoglyceride are presented in Table 3.

**Table 3:** Oil-binding capacity of single-component oleogels based on BW and MG.

Structurant Content, % (*w*/*w*)	Oil-Binding Capacity of BW Oleogel, %	Oil-Binding Capacity MG Oleogel, %
3%	61.20 ± 0.07 ^a^	58.53 ± 0.03 ^a^
6%	98.10 ± 0.04 ^b^	85.85 ± 0.04 ^b^
9%	99.28 ± 0.06 ^c^	89.90 ± 0.06 ^c^
12%	99.55 ± 0.03 ^c^	95.52 ± 0.05 ^d^

Note: Results are expressed as mean ± standard error of the mean (*n* = 3). Different letters indicate significant differences in the same column (*p* < 0.05).

The results obtained showed that in the oil blend studied, beeswax provided more effective retention of the liquid oil phase compared with monoglycerides. This is presumably due to the ability of beeswax components to form a well-developed, spatially connected crystal network that effectively restricts the mobility of the liquid oil phase. This is consistent with literature data according to which beeswax-based oleogels were characterized by a high oil-binding capacity [63]. At the same time, this increase should be regarded as system-dependent, since oil-binding capacity is determined not only by the type of structuring agent, but also by the composition of the oil, the concentration of the oleogelator, and the characteristics of the crystalline structure formed [44,64,65].

**Figure 3:**
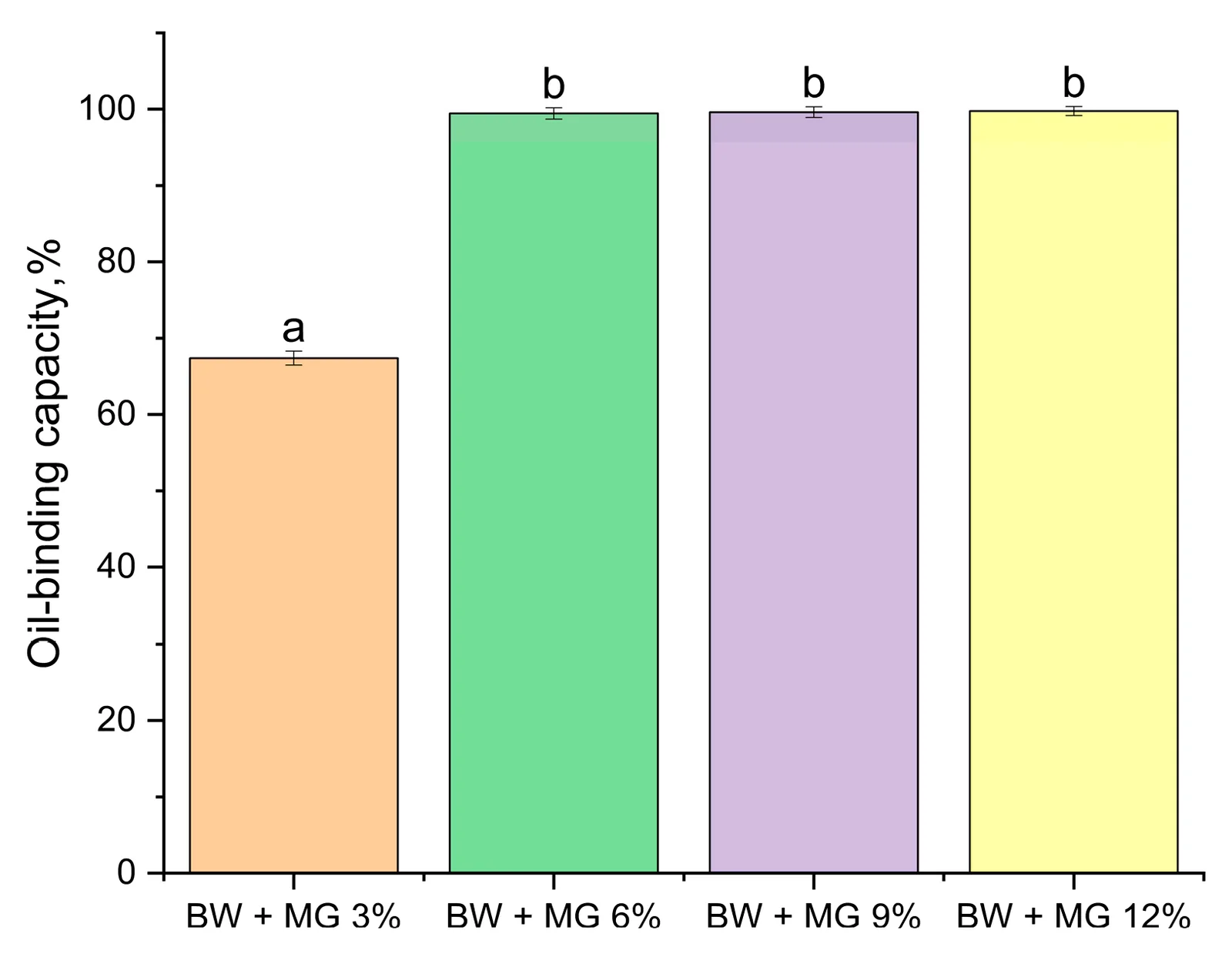
Oil-binding capacity of the oleogels. Values are presented as mean ± standard deviation (*n* = 3); different letters within the same structuring agent system indicate significant differences between concentrations (*p* < 0.05).

Figure 3 presents the oil-binding capacity values of oleogels obtained using the binary beeswax–monoglyceride (BW + MG, 1:1) structuring agent system at various total structuring agent concentrations.

Analysis of the oil-binding capacity revealed a substantial influence of the concentration of structuring agents on the ability of the oleogels to retain liquid oil. At a structuring agent content of 3%, the oil-binding capacity was 67.39%, which indicates an insufficiently formed crystalline network. An increase in the concentration to 6% led to a sharp rise in the indicator to 99.46%. A further increase in the structuring agent content to 9% and 12% was accompanied by an insignificant increase in the oil-binding capacity to 99.62% and 99.77%, respectively. The results obtained are consistent with the data of previously conducted studies, in which an increase in the concentration of structuring agents promoted an increase in the oil-binding capacity of oleogels [66].

Thus, the results obtained indicate that a structuring agent concentration of 6% is sufficient for the formation of a stable crystalline structure that effectively retains the liquid oil phase.

When the binary BW + MG (1:1) system was used, the oil-binding capacity of the oleogels was higher than that of the monoglyceride-based samples across the entire concentration range studied. Already at 3%, the OBC of the mixed oleogel reached 67.39%, exceeding the values of the single-component beeswax- and monoglyceride-based systems. With a further increase in concentration to 6–12%, the binary oleogels retained virtually the entire liquid oil phase, with oil-binding capacity values ranging from 99.46 to 99.77%.

At concentrations of 6–12%, the oil-binding capacity of the binary oleogels was close to that of the beeswax-based samples and markedly higher than that of the oleogels structured with monoglyceride alone. This suggests that, within the mixed system, beeswax was primarily responsible for retaining the liquid phase, whereas monoglyceride contributed to the formation of a mechanically stronger structure.

The obtained results on hardness and oil-binding capacity are consistent with previous studies, which have noted that the use of binary structuring agent systems, particularly monoglycerides combined with waxes or phytosterols, makes it possible to modify the properties of oleogels. This is because different structuring agents participate differently in shaping the structure of the oil phase: monoglycerides can promote the formation of more rigid lamellar structures, whereas waxes form a crystalline framework and improve the immobilization of liquid oil. As a result, such systems allow the hardness and oil-binding capacity of oleogels to be regulated [36,37,67].

Thus, the binary system makes it possible to obtain oleogels with high oil-binding capacity and sufficient hardness. From a technological standpoint, the 6% and 9% samples proved to be the most successful, as they provided high oil-binding capacity combined with moderate hardness.

### 3.3. Inverse Vial Test of Oleogels

**Figure 4:**
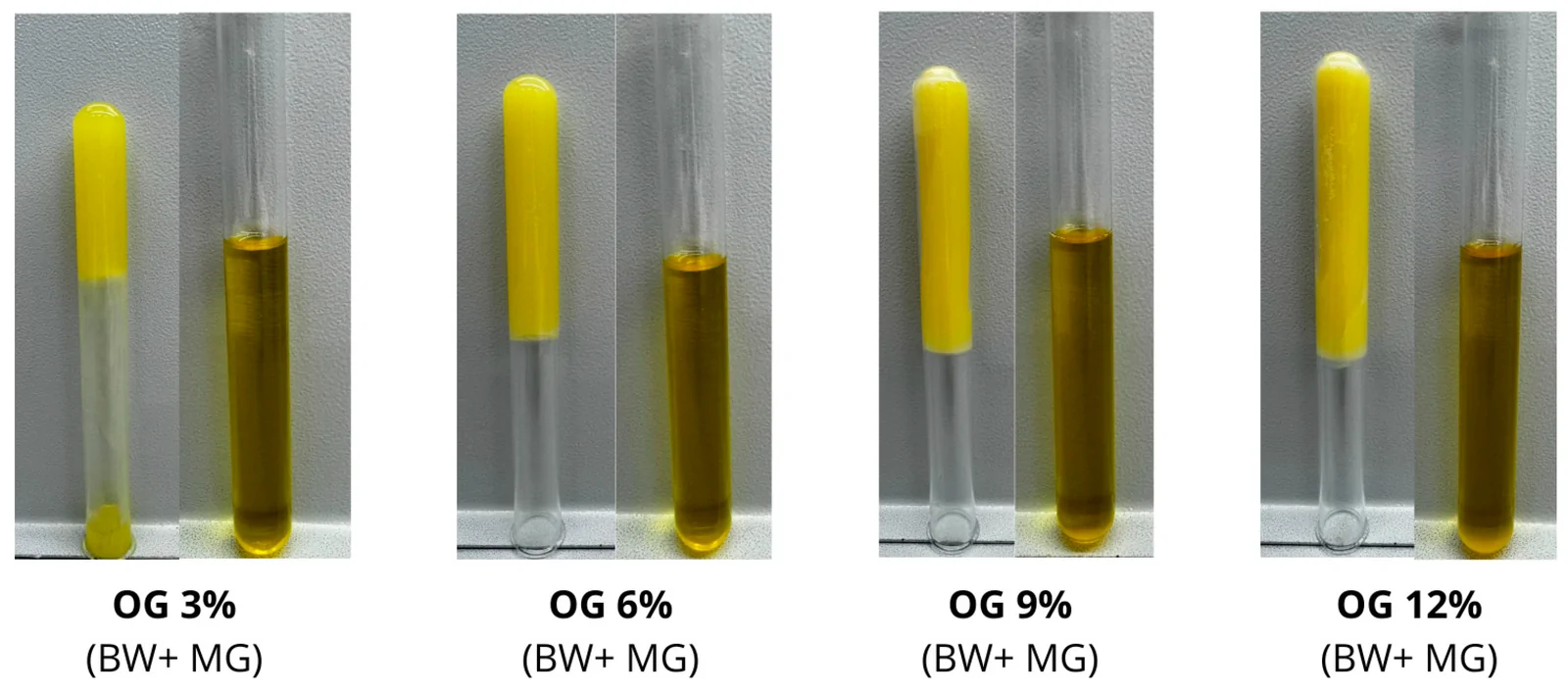
Oleogels based on blended oils with different concentrations of structuring agents (OG: Oleogel, BW: Beeswax, MG: Monoglyceride).

Figure 4 shows appearances of the oleogels obtained based on blended oils with different concentrations of binary structuring agents at 3%, 6%, 9% and 12% (*w*/*w*).

As a result of the visual inspection of the obtained oleogels, it was established that the 3% sample did not form a self-supporting structure and exhibited flowability when tilted, being characterized by incomplete structuring of the oil phase, whereas the 6%, 9% and 12% samples did not exhibit flowability when inverted, maintaining a stable solid state at 25.0 ± 0.5 °C. This indicates their stable structure and the possibility of their use in food systems where uniformity and stability of texture are important. As the concentration of structuring agents increased, the oleogels acquired a more intense color and were characterized by a decrease in transparency.

### 3.4. Thermal Properties of Oleogels

**Figure A1:**
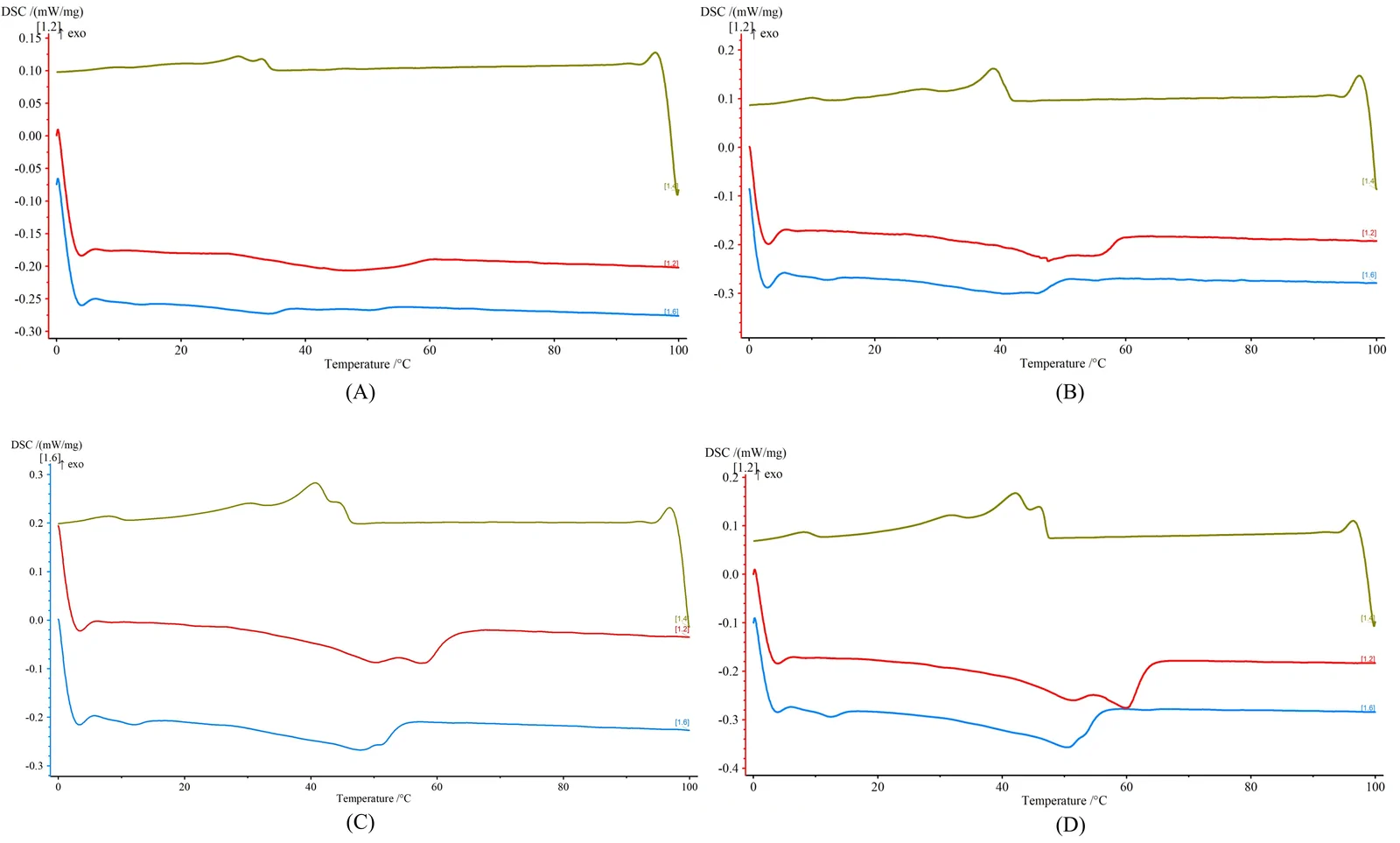
DSC curves of the obtained oleogels. (**A**) DSC thermograms OG 3% (BW + MG), (**B**) DSC thermograms OG 6% (BW + MG), (**C**) DSC thermograms OG 9% (BW + MG), and (**D**) DSC thermograms OG 12% (BW + MG).

DSC analysis was carried out to assess how the concentration of the binary structuring agent affects the thermal behavior of the oleogels obtained based on the blended vegetable oil. The melting parameters were determined from the second-heating curves, since this approach makes it possible to characterize the system after controlled cooling and the re-formation of the crystalline network. The melting characteristics of the oleogels with 3%, 6%, 9% and 12% binary structuring agent, according to the second-heating DSC data, are presented in Table 4, and the DSC curves are shown in Figure A1.

The oleogel with a 3% structuring agent content was distinguished by the least pronounced degree of structuring. On the second-heating curve, three endothermic transitions with maxima at 4.0 °C, 34.1 °C and 50.3 °C were recorded. At the same time, the high-temperature peak at 50.3 °C had a very low enthalpy (0.14 J/g), which indicates a small amount of stable crystalline fraction. The total melting enthalpy of the sample was 3.89 J/g and was the lowest among all the oleogels studied. During the cooling stage, the crystallization transitions were weakly pronounced, which is associated with the low concentration of the structuring agent and the formation of an insufficient gel network. Such a sample can be characterized as a soft, weakly structured lipid system.

When the structuring agent concentration was increased to 6%, the thermal profile became more pronounced. The main melting peak was observed at 40.71 °C, and its enthalpy was 2.76 J/g. The total melting enthalpy increased to 6.71 J/g, which indicates a growth of the organized crystalline phase compared with the 3% sample. On the cooling curve, a pronounced exothermic transition was noted in the region of 39–40 °C, associated with the crystallization of the structuring phase and the formation of the gel network. At the same time, the value of the total melting enthalpy shows that the oleogel structure became moderately crystallized, without signs of excessive densification. From a technological point of view, this is important, since such a system can provide sufficient stability while preserving the plasticity of the fat phase.

A further increase in the structuring agent concentration to 9% was accompanied by an intensification of the structuring, with the main melting peak increasing to 47.90 °C and its enthalpy rising to 7.60 J/g. The total melting enthalpy reached 12.42 J/g, which is higher than in the previous oleogel samples, indicating a noticeable increase in the amount of stable crystalline phase. At the cooling stage, more intense exothermic transitions were observed for this sample, which confirms the active crystallization of beeswax and monoglyceride in the oil phase. The most pronounced degree of structuring was observed in the oleogel with 12% binary structuring agent. The main melting peak of this sample was at 50.51 °C, and the enthalpy of the main transition was 11.08 J/g, which is the maximum value among the oleogel samples studied. The total melting enthalpy reached 13.74 J/g, confirming the formation of a dense and more thermostable crystalline network. The most pronounced exothermic transitions were also observed on the cooling curve, which indicates an intense crystallization of the structuring phase.

Previously conducted studies have established that the binary structuring agent system provides a more stable organization of the lipid matrix, intensifying the endothermic transitions and increasing the total melting enthalpy of the oleogels [29,68].

The DSC analysis results showed that at a binary structuring agent content of 3%, the thermal transitions were weakly pronounced. This may be related to limited formation of an ordered crystalline structure in the oil phase. Despite the presence of a high-temperature melting peak, its contribution to the overall thermal effect was minor, as evidenced by the low melting enthalpy value. When the structuring agent content was increased to 12%, the thermal effects became more pronounced, as confirmed by the increase in total melting enthalpy, indicating a more developed crystalline structure. This observed pattern is consistent with the findings of Jeong and Oh, who established that increasing the content of the beeswax–glycerol monostearate binary system from 5 to 10% was accompanied by an increase in the melting peak temperature [36]. However, for spreads, an excessive intensification of crystallization processes is not always desirable, as it would lead to a decrease in plasticity and impair spreadability.

The oleogels with 6% and 9% structuring agent occupied an intermediate position. According to the DSC data, these samples were characterized by more pronounced thermal effects and higher total melting enthalpy values compared to the 3% oleogel, but were inferior to the 12% sample in terms of the degree of crystalline phase development. These findings are of practical importance for spread development, since such products do not require maximum hardness, but rather an optimal combination of structural stability and good spreadability [58].

From a technological standpoint, the most promising sample was the one containing 6% (*w*/*w*) of the binary structuring agent system, which was characterized by optimal combination of thermal properties and hardness.

### 3.5. Microstructural Properties of Oleogels

The microstructure of the oleogels was examined in order to establish the effect of the binary structuring agent concentration on the morphology and distribution of the crystals within the oil phase of the oleogel. The morphology of the crystals formed in the oleogels exerts a substantial influence on their structural properties and, consequently, on the characteristics of the food products in which they are incorporated [69].

**Figure 5:**
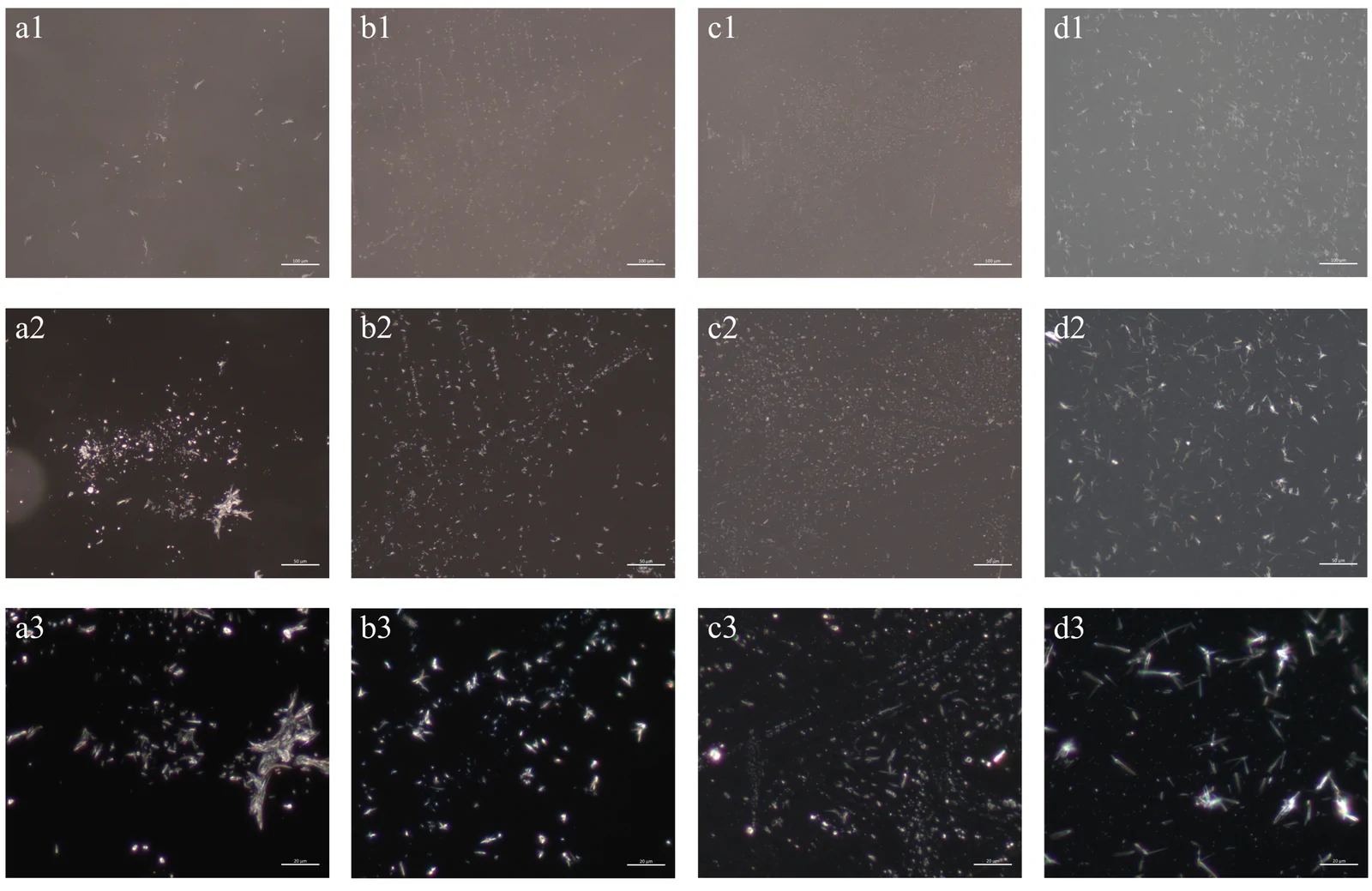
Micrographs of the oleogels prepared using different structuring agents, in EPI DF mode. (**a1**–**a3**) OG 3% (BW + MG); (**b1**–**b3**) OG 6% (BW + MG); (**c1**–**c3**) OG 9% (BW + MG); (**d1**–**d3**) OG 12% (BW + MG).

The microstructures of the oleogel samples obtained based on the blended oil with different concentrations of structuring agents of 3%, 6%, 9% and 12% are presented in Figure 5.

In the images shown in Figure 5, the light areas represent crystalline particles, while the dark zones represent liquid blended oil. As can be seen from the obtained micrographs, an increase in the concentration of the binary structuring agent system altered the number, distribution and morphology of the crystals. At a concentration of 3% (*w*/*w*), sparse and unevenly distributed crystalline formations were observed, whereas at 6 and 9% (*w*/*w*), the number of small crystals increased and the uniformity of their distribution within the oil phase improved. At a structuring agent concentration of 12% (*w*/*w*), coarsening and elongation of the crystalline formations were observed, accompanied by the appearance of a pronounced needle-like morphology, as shown in Figure 4. These changes were accompanied by an increase in the hardness of the oleogel and may indicate enhanced structuring of the oil phase.

The combined use of beeswax and monoglycerides resulted in the formation of a heterogeneous crystal structure comprising needle-like crystals and small aggregated formations. A similar combination of crystals of different morphology was reported by Li et al. [70], who observed comparable crystal morphologies when a monoglyceride was combined with a wax in an oleogel based on high-oleic sunflower oil.

According to previously conducted studies of Doan et al. [71], the chemical properties of waxes substantially influence the morphology of the crystals, forming needle-like crystals, which is associated with the predominance of wax esters present in waxes.

The crystalline structures formed by beeswax exhibited a fine needle-like morphology and relatively small crystal dimensions, which may be attributed to the complex chemical composition of beeswax, predominantly composed of wax esters [71,72]. Monoglycerides distribute readily throughout the oil phase and actively participate in oleogel network formation. This allows liquid oil to be retained more effectively within the crystalline matrix, resulting in a more stable oleogel overall. Monoglycerides are known to produce snowflake- and spherulite-shaped crystal morphologies [67].

It should also be noted that the formation of the crystal structure of oleogels is influenced not only by interactions between the structuring agents, but also by the composition of the oil. Thus, Dimakopoulou-Papazoglou et al. examined binary systems of waxes, including beeswax, and monoglycerides in various vegetable oils, and established that needle-like, platelet-like and spherulitic structures were formed in different oils. The authors concluded that crystal morphology is determined not only by the combination of structuring agents, but also by the composition of the liquid oil phase, which affects the compatibility of the oil phase, the wax and the monoglycerides [29].

Thus, in the oleogels structured with a mixture of beeswax and monoglycerides, small crystalline formations were observed alongside needle-like crystals, the size and prominence of which changed with increasing structuring agent concentration. At a higher content of the binary system, the needle-like crystals became larger and formed a more developed crystal structure. The observed morphology may be associated with the combined influence of beeswax and monoglycerides on crystallization processes and is consistent with the increase in hardness of the oleogels.

### 3.6. Infrared Spectroscopy of Oleogels

**Figure A2:**
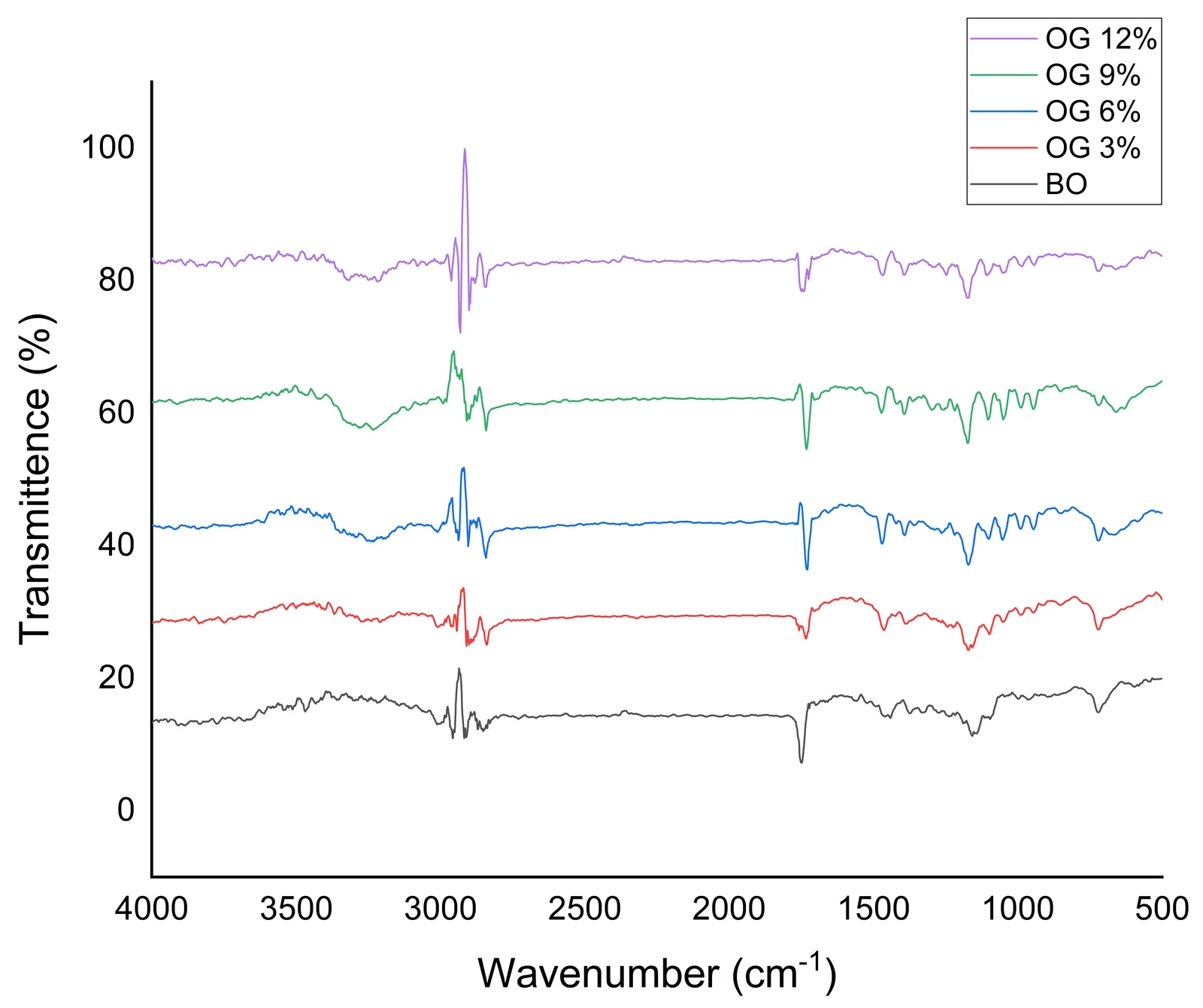
FTIR spectra of the blended oil and of the oleogels with different concentrations of structuring agents.

To assess the possible molecular interactions in the oleogel system, FTIR analysis of the blended vegetable oil and of the oleogels structured with the binary beeswax–monoglyceride system in a ratio of 1:1 at concentrations of 3%, 6%, 9% and 12% was carried out, as shown in Figure A2.

The FTIR spectra (Figure A2) of the original blended oil and of the oleogels were characterized by a similar set of main absorption bands, which indicates the preservation of the chemical structure of the lipid phase after structuring. In the spectra of all samples, intense bands were observed in the region of about 2920 and 2850 cm^−1^, corresponding to the asymmetric and symmetric stretching vibrations of the -CH_2_- groups in the aliphatic chains of the fatty acids [36]. The pronounced band observed in the region of 1740–1745 cm^−1^ is associated with the stretching vibrations of the carbonyl group C=O of the ester bonds of the triacylglycerides, which confirms the preservation of the main structure of the vegetable oils. The bands in the region of 1460 cm^−1^ can be attributed to the deformation vibrations of the CH_2_ and CH_3_ groups, whereas the peaks in the region of 1160–1100 cm^−1^ are associated with the stretching vibrations of the C-O and C-O-C bonds of the ester groups. The band at about 720 cm^−1^ corresponds to the rocking vibrations of the long methylene chains and is characteristic of lipid systems with long-chain aliphatic fragments.

The addition of the binary structuring agent did not lead to the appearance of new absorption bands or to the disappearance of the characteristic peaks of the blended oil. This indicates that, during the formation of the oleogel, no new covalent bonds were formed and the chemical nature of the lipid phase remained unchanged. At the same time, as the structuring agent concentration increased from 3% to 12%, a more pronounced manifestation of the bands associated with the aliphatic chains was observed, especially in the regions of about 2920, 2850 and 720 cm^−1^. This may be associated with the increase in the proportion of beeswax and monoglyceride, which contain long-chain hydrocarbon fragments, as well as with the strengthening of the ordering of the lipid structure.

The FTIR spectra confirm that the structure of the oleogels was formed predominantly through physical interactions between the components of the system, including van der Waals interactions between the aliphatic chains, hydrogen bonds involving the functional groups of the monoglyceride and beeswax, and the crystallization of the structuring phase in the oil matrix [73].

The absence of new bands indicates the physical nature of the structuring, whereas the strengthening and slight changes in the intensity of individual bands with increasing structuring agent concentration reflect the increase in the degree of structuring of the oleogel system.

### 3.7. Apparent Viscosity

**Figure 6:**
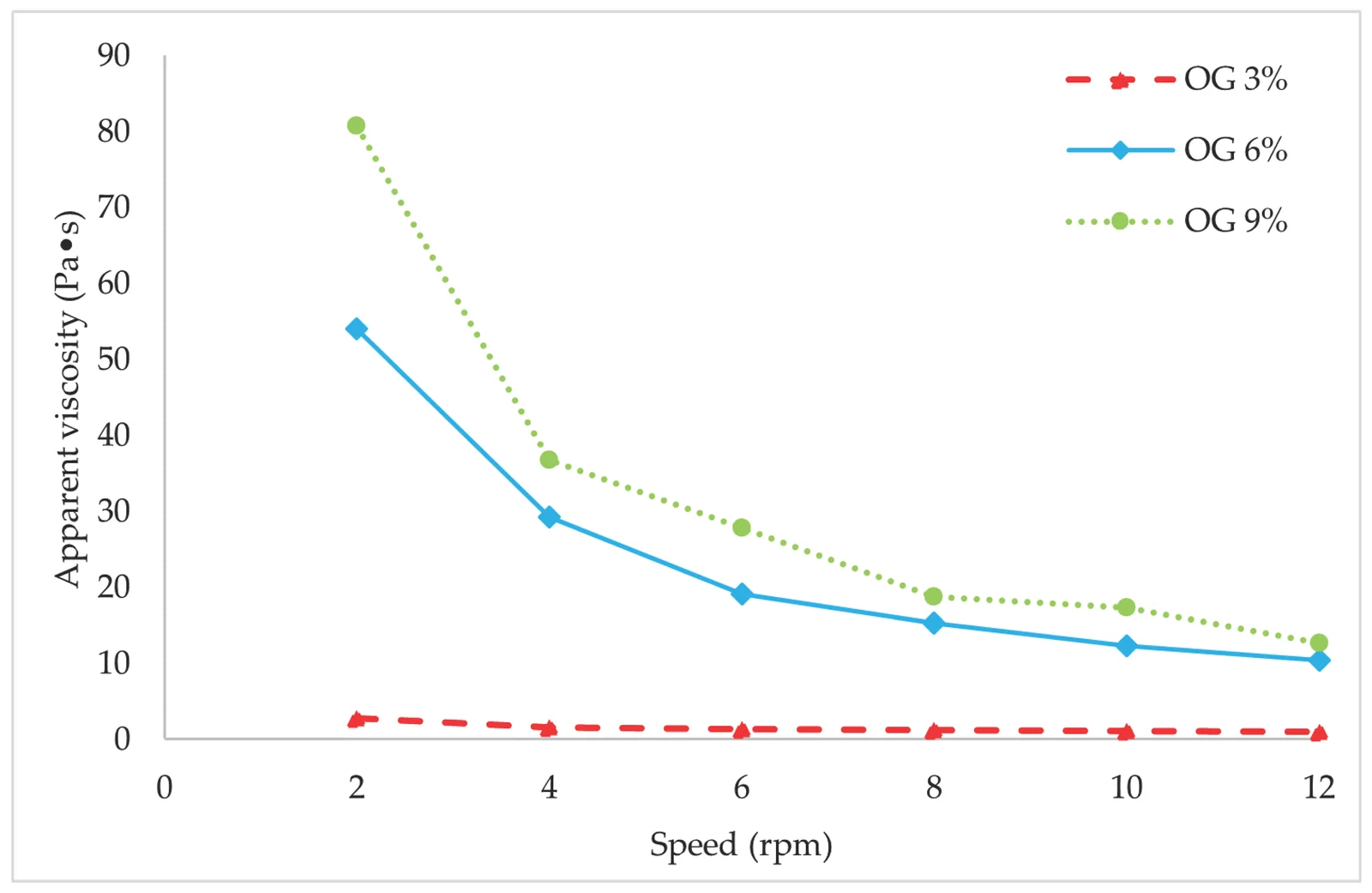
Dependence of the apparent viscosity of oleogels with different contents of the binary BW-MG structuring agent system on the spindle rotational speed. Red line stands for OG 3%, blue for OG 6%, and green is for OG 9%.

Figure 6 presents the dependence of the apparent viscosity of the OGs containing 3, 6 and 9% (*w*/*w*) of the binary BW + MG structuring agent system on the spindle rotational speed. For all the samples studied, an increase in rotational speed was accompanied by a decrease in apparent viscosity. Such behavior is characteristic of non-Newtonian structured systems and indicates a pronounced tendency towards shear thinning.

For the OG 3% (*w*/*w*) sample, the apparent viscosity decreased from 2.75 ± 0.14 Pa·s at 2 rpm to 0.98 ± 0.01 Pa·s at 12 rpm, that is, by 64.40%. For the OG 6% (*w*/*w*) sample, the value decreased from 54.10 ± 3.98 to 10.39 ± 0.09 Pa·s, corresponding to a reduction of 80.80%. For OG 9% (*w*/*w*), the viscosity decreased from 80.77 ± 5.95 Pa·s at 2 rpm to 12.71 ± 3.06 Pa·s at 12 rpm, or by 84.70%. The most pronounced decrease in viscosity was observed as the rotational speed increased from 2 to 4 rpm. Within this range, the viscosity of OG 3% (*w*/*w*) decreased by 41.70%, that of OG 6% (*w*/*w*) by 45.90%, and that of OG 9% (*w*/*w*) by 54.50%. With a further increase in speed, the decrease became more gradual. Presumably, the initial mechanical action caused preferential disruption of the weakest intracrystalline contacts and of the larger aggregates, whereas at higher speeds the flow was governed by the more stable part of the crystal network. A similar dependence was established by Zhang and Xu et al. [74]. For oleogels based on saturated monoglycerides, the apparent viscosity of all samples decreased with increasing shear rate, which the authors attributed to disruption of the gel network. At the same time, increasing the monoglyceride content from 4 to 10% raised the viscosity, owing to the greater number of crystals, densification of the three-dimensional structure, and enhanced retention of liquid oil.

At the same rotational speed, the apparent viscosity increased consistently with increasing content of the binary BW + MG structuring agent system. Thus, at 2 rpm, the viscosity of OG 6% (*w*/*w*) was approximately 19.70 times higher than that of OG 3% (*w*/*w*), whereas the transition from 6 to 9% (*w*/*w*) was accompanied by a further increase in viscosity of 49.30%. Throughout the entire speed range, the sequence OG 9% (*w*/*w*) > OG 6% (*w*/*w*) > OG 3% (*w*/*w*) was maintained.

At 10 rpm the differences between the samples decreased somewhat; nevertheless, the 9% (*w*/*w*) oleogel still exhibited the highest resistance to spindle rotation. This indicates that an increase in the total content of the binary structuring agent system increased the volume fraction of the crystalline phase and the number of contacts between structural elements restricting the mobility of the oil phase. The observed pattern is consistent with the data of Zhang and Xu et al. [74], according to which an increase in MG concentration is accompanied by the formation of a denser network of small needle-like crystals and by improved rheological and mechanical characteristics of the oleogels. The authors also associate the structuring of MG systems with the self-assembly of polar glycerol groups, the formation of hydrogen bonds, and the subsequent crystallization of the hydrocarbon chains.

In the study by Zhang and Geng et al. [75] on oleogels based on a mixture of ferulic acid and monoglycerides, an increase in the total structuring agent content from 4 to 5% likewise resulted in a higher apparent viscosity. The authors attributed this to the formation of a greater number of crystals and a more complete spatial network.

For systems directly containing beeswax and monoglycerides, ref. [37] established a pseudoplastic flow behavior and showed that the BW-to-MG ratio substantially affects the rheological parameters of the oleogel. The flow curves obtained by the authors were described by the Herschel–Bulkley model, which confirms the presence of a structured network that is progressively disrupted under shear. A study of mixed BW-MG oleogels likewise showed that the interaction between beeswax crystals and MG can reinforce the overall structure of the oleogel [36].

OG 12% (*w*/*w*) was not included in the graphical dependence, since its high consistency caused the viscometer readings to exceed the upper limit of the measuring range at the selected combination of spindle and rotational speed. The appearance of the EEEEE message indicated overloading of the measuring system, that is, the resistance of the sample to spindle rotation exceeded the upper limit of the instrument measuring range.

Thus, the oleogels containing 6 and 9% (*w*/*w*) of the binary structuring agent system exhibited the most balanced rheological properties. The OG 6% (*w*/*w*) sample possessed a moderately high apparent viscosity and retained a more pronounced plasticity, which may facilitate its mixing and distribution over a surface. OG 9% (*w*/*w*) was distinguished by a higher viscosity and structural stability, indicating better retention of the liquid oil phase and greater resistance to mechanical action. By contrast, the 3% (*w*/*w*) sample was characterized by an insufficiently developed structure, whereas the 12% (*w*/*w*) oleogel proved excessively thick and exceeded the measuring range of the viscometer. At low intensities of mechanical action, the more concentrated oleogels retained a high viscosity and a stable structure, whereas during mixing or spreading, their viscosity decreased, facilitating distribution of the product.

## 4. Conclusions

The conducted studies confirmed the feasibility of structuring the developed vegetable oil blend, comprising high-oleic sunflower, safflower, and flaxseed oils in a 60:30:10 ratio, using a binary beeswax–monoglyceride structuring system. The resulting blend was characterized by a more balanced fatty acid composition and an ω-6/ω-3 ratio of 6:1, which falls within the recommended range and confirms its potential as a lipid base.

It was established that increasing the total concentration of the binary structurant system was accompanied by enhanced oleogel structuring, increased hardness and oil-binding capacity, and changes in thermal, microstructural, and rheological characteristics. At concentrations of 6 and 9% (*w*/*w*), the binary oleogels exhibited higher hardness compared to the corresponding single-component systems, indicating a more pronounced structuring effect from the combined use of beeswax and monoglycerides within this concentration range.

A comprehensive evaluation of textural, oil-binding, microstructural, thermal, and rheological characteristics made it possible to identify an optimal total concentration of the binary structurant system at 6% (*w*/*w*). At this concentration, a balanced combination of hardness, oil-binding capacity, thermal characteristics, and rheological behavior was achieved with a relatively low structurant content. The resulting oleogel may be regarded as a potential fat base for further spread development. However, confirming its technological suitability will require studies in model and finished formulations, including instrumental assessment of spreadability, sensory characteristics, and the dynamics of oxidative changes during storage.

## Figures and Tables

**Table 1:** Fatty acid composition of the original vegetable oils and the blended oil.

Fatty Acid	Oil Sample			
	HOSO	SFO	LO	BO
C16:0	4.34 ± 0.02 ^d^	6.67 ± 0.10 ^b^	8.95 ± 0.31 ^a^	5.57 ± 0.23 ^bc^
C14:0	0.08 ± 0.01 ^b^	0.17 ± 0.02 ^a^	nd	0.09 ± 0.03 ^b^
C18:0	5.21 ± 0.09 ^b^	2.61 ± 0.03 ^c^	6.28 ± 0.03 ^a^	4.67 ± 0.64 ^b^
C20:0	0.38 ± 0.04 ^a^	0.25 ± 0.03 ^b^	0.29 ± 0.04 ^a^	0.31 ± 0.06 ^a^
C24:0	0.44 ± 0.04 ^a^	0.18 ± 0.02 ^c^	nd	0.33 ± 0.03 ^b^
C16:1	0.08 ± 0.04 ^a^	0.16 ± 0.03 ^a^	0.10 ± 0.02 ^a^	0.07 ± 0.03 ^b^
C18:1(cis-9)	80.29 ± 0.79 ^a^	11.84 ± 0.09 ^d^	17.60 ± 0.19 ^c^	53.32 ± 0.39 ^b^
C20:1(cis-11)	0.35 ± 0.03 ^a^	nd	0.29 ± 0.03 ^a^	0.24 ± 0.04 ^b^
C18:2n6c	8.12 ± 0.04 ^d^	77.42 ± 0.35 ^a^	15.84 ± 0.66 ^c^	29.81 ± 0.49 ^b^
C18:3n3	0.18 ± 0.03 ^c^	0.09 ± 0.03 ^c^	49.92 ± 0.85 ^a^	4.78 ± 0.20 ^b^
Σ SFA	10.45 ^b^	9.88 ^b^	15.52 ^a^	10.97 ^b^
Σ MUFA	80.72 ^a^	12.0 ^d^	17.99 ^c^	53.63 ^b^
Σ PUFA	8.30 ^d^	77.51 ^a^	65.76 ^b^	34.59 ^c^
Ratio ω-6: ω-3	45:1	860:1	0.32:1	6:1
MUFA/SFA	7.72 ^a^	1.21 ^c^	1.15 ^c^	4.88 ^b^
COX	1.68 ^d^	8.11 ^b^	12.59 ^a^	4.64 ^c^

Note: Results are expressed as mean ± standard error of the mean (*n* = 3). Different letters indicate significant differences within the row (*p* < 0.05). HOSO: high-oleic sunflower oil; SFO: safflower oil; LO: linseed oil; BO: blended oil; ω-6: omega-6 fatty acids (ω-6 FAs); ω-3: omega-3 fatty acids (ω-3 FAs); MUFA: monounsaturated fatty acid; PUFA: polyunsaturated fatty acid; SFA: saturated fatty acid; COX: calculated oxidizability value; nd: not detected.

**Table 2:** Hardness of single-component oleogels structured with beeswax (BW) and monoglycerides (MGs).

Structurant Content, % (*w*/*w*)	BW Hardness, N	MG Hardness, N
3%	0.08 ± 0.018 ^a^	1.02 ± 0.04 ^a^
6%	0.97 ± 0.13 ^b^	1.35 ± 0.03 ^b^
9%	1.45 ± 0.05 ^c^	2.93 ± 0.05 ^c^
12%	4.75 ± 0.04 ^d^	6.28 ± 0.03 ^d^

Note: Results are expressed as mean ± standard error of the mean (*n* = 3). Different letters indicate significant differences in the same column (*p* < 0.05).

**Table 4:** Melting characteristics of the oleogels with different concentrations of structuring agents according to the second-heating DSC data.

Thermal Transition	OG 3% (BW + MG)	OG 6% (BW + MG)	OG 9% (BW + MG)	OG 12% (BW + MG)
Tm, peak1, °C	4.00 ± 1.51 ^a^	2.90 ± 0.27 ^a^	3.40 ± 0.26 ^a^	3.90 ± 0.17 ^a^
ΔHm, J/g	3.26 ± 0.09 ^b^	3.80 ± 0.15 ^c^	4.33 ± 0.07 ^d^	2.11 ± 0.02 ^a^
Tm, peak2, °C	34.10 ± 0.95 ^b^	12.50 ± 0.79 ^a^	12.05 ± 0.81 ^a^	12.50 ± 0.08 ^a^
ΔHm2, J/g	0.49 ± 0.14 ^b^	0.15 ± 0.02 ^a^	0.49 ± 0.04 ^b^	0.55 ± 0.01 ^b^
Tm, main, °C	50.30 ± 1.55 ^b^	40.71 ± 1.22 ^a^	47.90 ± 1.35 ^b^	50.51 ± 0.02 ^b^
ΔHm, main, J/g	0.14 ± 0.02 ^a^	2.76 ± 0.21 ^b^	7.60 ± 0.34 ^c^	11.08 ± 0.03 ^d^
ΔHm, total, J/g	3.89 ± 0.32 ^a^	6.71 ± 0.15 ^b^	12.42 ± 0.08 ^c^	13.74 ± 0.01 ^d^
Cp, J/(g·K)	2.42 ± 0.04 ^b^	2.32 ± 0.14 ^ab^	2.74 ± 0.02 ^c^	2.19 ± 0.01 ^a^

Note: Results are expressed as mean ± standard error of the mean (=3). Different letters indicate significant differences within the row (*p* < 0.05).

## Data Availability

The original contributions presented in the study are included in the article, further inquiries can be directed to the corresponding author.

## Abbreviations

The following abbreviations are used in this manuscript:

BW	Beeswax
MG	Monoglyceride
BW + MG	Binary beeswax–monoglyceride structuring agent system
OG	Oleogel
HOSO	High-oleic sunflower oil
SFO	Safflower oil
LO	Linseed oil
BO	Blended oil
SFA	Saturated fatty acid(s)
MUFA	Monounsaturated fatty acid(s)
PUFA	Polyunsaturated fatty acid(s)
TFA	Trans fatty acid(s)
ω-6/ω-3 (n-6/n-3)	Omega-6/omega-3 fatty acids
COX	Calculated oxidizability value
AI	Atherogenic index
TI	Thrombogenic index
WHO	World Health Organization
FAO	Food and Agriculture Organization
LA/ALA	Linoleic acid/alpha-linolenic acid
GC-MS	Gas chromatography–mass spectrometry
FAME	Fatty acid methyl ester(s)
rpm	Revolutions per minute
OBC	Oil-binding capacity
DSC	Differential scanning calorimetry
ATR	Attenuated total reflectance
FTIR	Fourier-transform infrared spectroscopy
EPI DF	Epi-illumination dark-field (microscopy)
ANOVA	Analysis of variance
